# Case Report: Extrapelvic Endometriosis in the Medial Thigh

**DOI:** 10.3389/frph.2021.692249

**Published:** 2021-07-22

**Authors:** Erica Pascoal, Stacey Rogers, Mathew Leonardi, Nicholas Leyland

**Affiliations:** Department of Obstetrics and Gynecology, McMaster University, Hamilton, ON, Canada

**Keywords:** extrapelvic, endometriosis, pathophysiology, musculoskeletal, surgery

## Abstract

Extrapelvic endometriosis, although rare, can present in the musculoskeletal system of reproductive-age women and cause significant pain and morbidity. The pathophysiology of this disease is not well understood. In this study, we described the case of a 39-year-old women with an inner-thigh mass causing catamenial pain. Core-biopsy of the mass confirmed endometriosis and she was referred to minimally-invasive gynecology for management. Surgical excision was performed by a multidisciplinary team and she remains pain-free postoperatively on hormonal therapy. Unique to this case, the patient had a history of pelvic fracture. Through this case report, we discuss possible pathophysiologic mechanisms of extrapelvic musculoskeletal endometriosis including the stem/progenitor cell theory and the role that musculoskeletal trauma may have in the development of this condition. Gynecologists play an important role in the recognition, diagnosis, and management of musculoskeletal endometriosis.

## Introduction

Endometriosis is a gynecologic condition associated with pelvic pain and infertility affecting approximately 10% of reproductive-age women and girls worldwide (1). Endometriosis is defined as the presence of endometrial-like tissue outside of the uterus with associated fibrosis and inflammatory reaction (1). Although typically confined to the pelvis, endometriosis can be found elsewhere, including the musculoskeletal system. Patients with musculoskeletal endometriosis have been found to present with cyclic pain and paresthesia according to the site of involvement (2). In a systematic review of 18 cases of musculoskeletal and central nervous system endometriosis, 61% of patients reported no dysmenorrhea or other clinical findings to suggest pelvic endometriosis (2), suggesting that musculoskeletal endometriosis can develop in the absence of clinically evident pelvic disease.

Preoperative investigation of musculoskeletal endometriosis has generally involved a combination of surgical biopsy and imaging. Although preoperative diagnosis is helpful, core biopsy has not always been conclusive in previously described cases (3, 4). Furthermore, MR imaging appearance of musculoskeletal endometriosis is variable as endometriosis tissue undergoes cyclic degeneration, and proliferation and appearance may change based on lesion age. Reported cases describe both hypointensity (4, 5) and hyperintensity (6, 7) on T2 weighted MR images. Preventing delay in treatment requires an index of suspicion and knowledge of presenting symptoms as excisional biopsy may be required for definitive diagnosis.

The pathogenesis of extrapelvic musculoskeletal endometriosis is not well understood. In this study, we present a rare case of endometriosis found in the medial thigh in a patient with a history of pelvic fracture. Through this report, we explore the possibility of previous pelvic disturbance, in the form of trauma or surgery, as an inciting event for the development of endometriosis in the lower limb.

## Case Description

A 39-year-old nulliparous woman presented to the emergency department with acute-on-chronic left groin pain. She was found to have a left-sided Bartholin's abscess, which was incised and drained, and a Word catheter was placed. The patient had a recurrence of the Bartholin's abscess 1 month later and was referred to a general gynecologist. On examination, there was a separate area of induration in the medial left thigh. A tender mobile mass was palpated 2 cm below the left vulva. On inquiry, the patient explained that this mass had been present >4 years and had become progressively more uncomfortable. She complained of fluctuating pain and swelling of the mass during her menstrual cycles, which was debilitating at times and caused difficulty with sitting and functioning at her desk job. She had a history of worsening dysmenorrhea although she had no radiologic evidence or pathologically confirmed history of endometriosis. The patient had sustained a left pubic rami fracture during a sporting accident approximately 15 years prior. The diagnosis of this fracture was delayed, and the fracture was ultimately managed conservatively. Otherwise, past medical history was remarkable for hypertension and 25-pack-year smoking history. She had previously been taking an oral contraceptive pill (OCP) for several years for contraceptive purposes, although this had been discontinued approximately 4 months before being seen by the team. There was a family history of endometriosis in both the mother and sister of the patient.

## Diagnostic Assessment and Management

**Figure 1:**
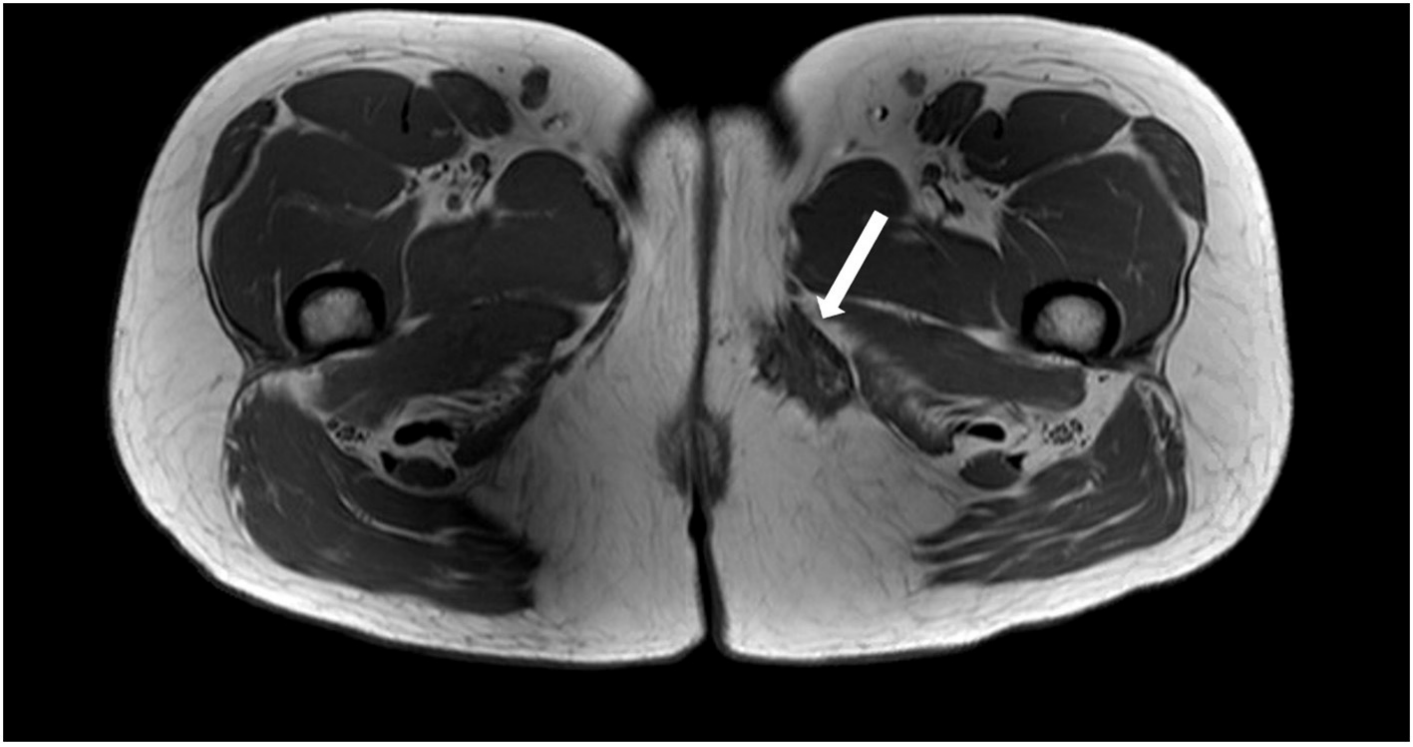
Axial T1 weighted MRI image demonstrating a spiculated hypointense lesion of the left medial thigh.

To better characterize the inner thigh mass, an MRI of the pelvis was performed. This demonstrated a left-sided vulvar collection consistent with a Bartholin's abscess and a separate lesion measuring 3.9 × 2.4 × 2.9 cm in the medial left thigh, inferior to the pubic ramus, near the posterior margin of the gracilis muscle. The lesion had spiculated borders and was predominantly T1 and T2 hypointense, although multiple punctate foci of T1 hyperintensity were seen within, consistent with hemorrhagic/proteinaceous foci (Figure 1). On T1-weighted MRI images, the fluid appears hypointense (dark) and fat appears hyperintense (bright), while both fluid and fat appear hyperintense on T2-weighted images. Muscle tends to demonstrate an intermediate intensity on both T1 and T2 images. An ultrasound-guided core biopsy of the mass was performed as an outpatient, and pathology concluded endometriosis. In follow-up, Bartholin's abscess of the patient resolved, however, her severe catamenial thigh pain persisted.

**Figure 2:**
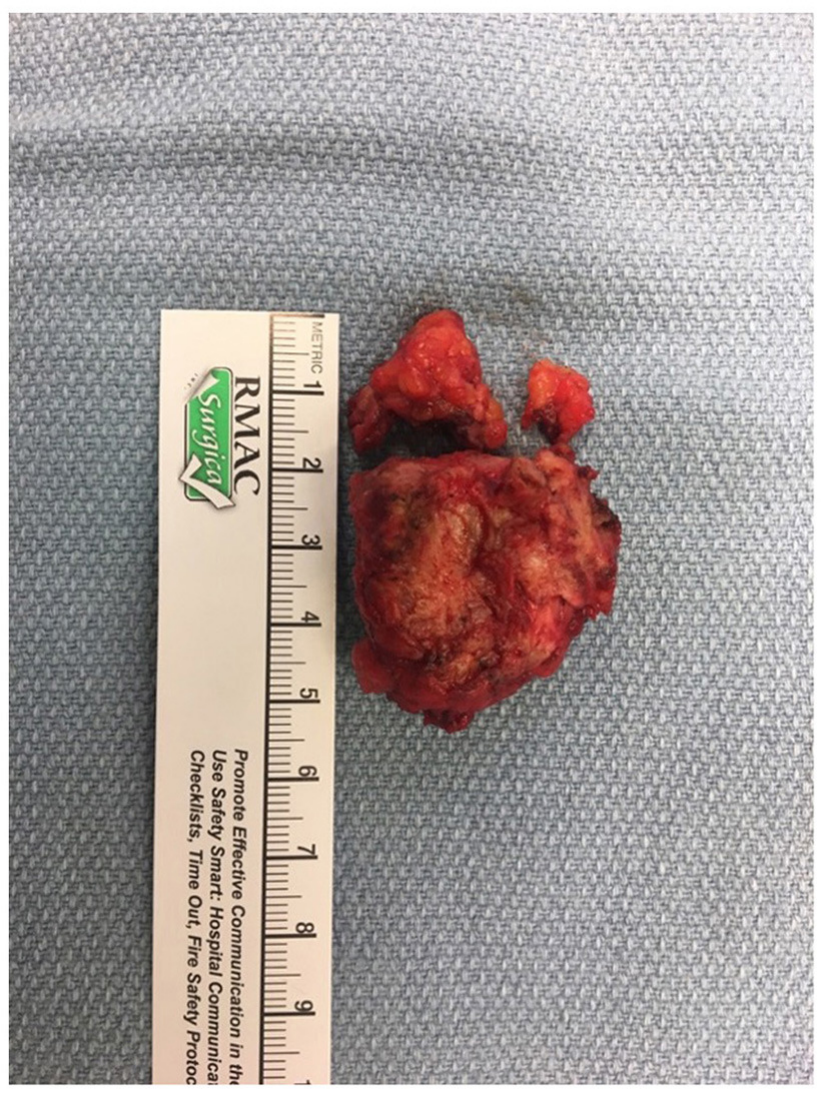
Timeline of the episode of cares from initial pelvic trauma to most recent follow-up.

**Figure 3:**
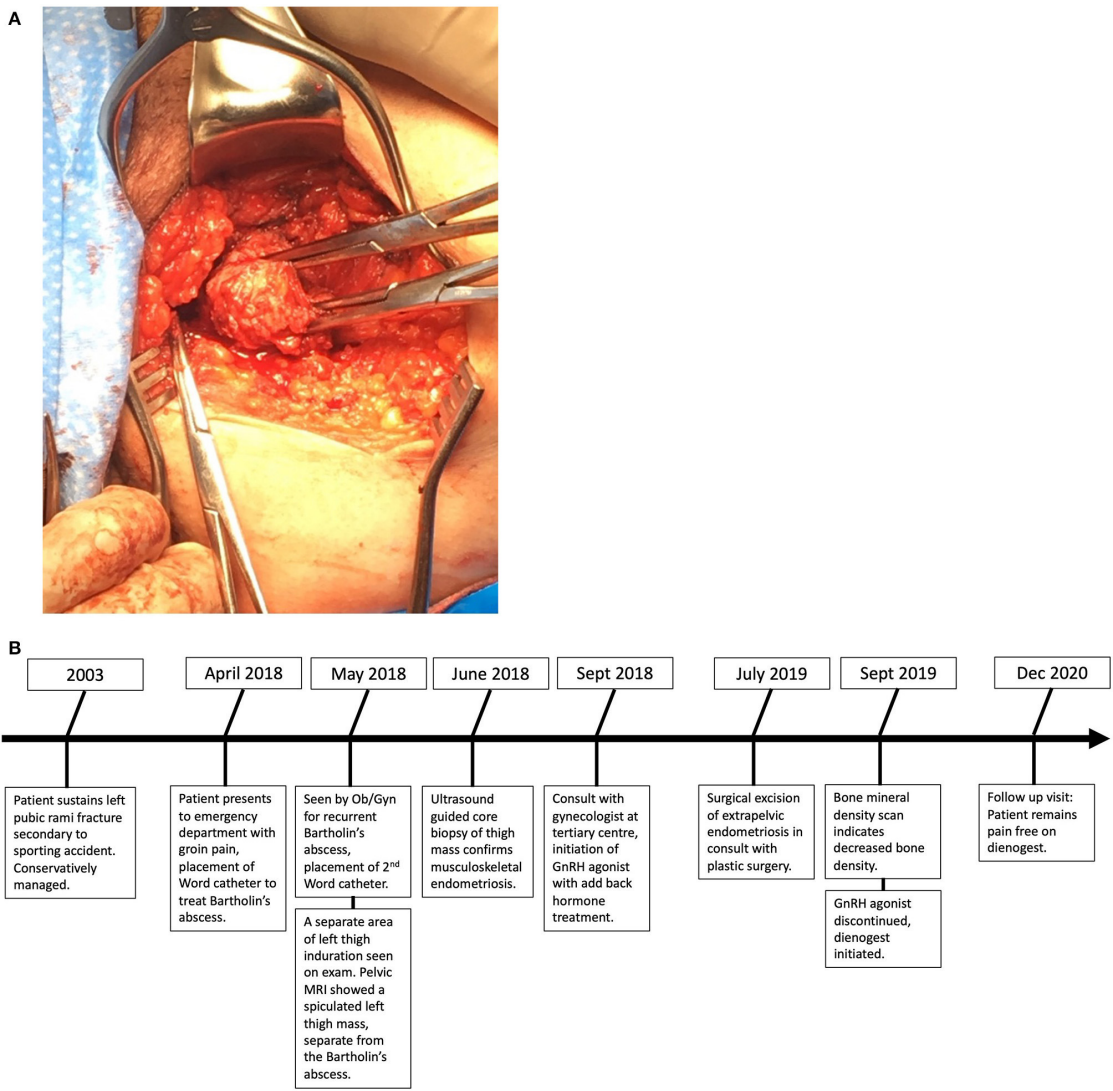
**(A)** Intraoperative dissection of medial thigh compartment, highlighting the endometriotic lesion in the center. **(B)** Retrieved operative specimens.

The patient was referred to an interdisciplinary minimally invasive gynecologic team at a tertiary center. She was initiated on medical management with gonadotropin-releasing hormone (GnRH) agonist therapy with add-back hormone replacement (Leuprolide acetate 11.25 mg intramuscular q 3 monthly and Norethindrone acetate 5 mg oral daily). Six months later, following two doses of Leuprolide acetate, she was seen in follow-up and had no significant level of improvement in symptoms. A decision was made to proceed with surgery, and she underwent surgical resection by gynecology in-consult with plastic surgery approximately 14 months following initial presentation (Figure 2). The patient had a radical dissection of endometriosis in the vulva, ischiorectal space, pubic ramus, and adductor muscles of the left leg. The mass was dissected off the insertion of the gracilis and the adductor magnus muscles and dissection extended into the ischiorectal fossa and vulva similar to a modified radical vulvectomy. This extensive dissection was required given the size of the lesion and in aim to achieve negative margins. Two specimens were retrieved measuring 2.3 and 3.2 cm in maximal dimensions (Figure 3). Histopathology of the mass lesions demonstrated fibrovascular tissue with multiple areas of endometriosis. Some of the endometrial glands appeared cystically dilated and contained calcified material, while others were smaller and showed hemorrhagic stroma surrounding them.

The postoperative course of the patient was complicated by wound infection and dehiscence requiring antibiotic treatment and wound packing to allow closure by secondary intent. Three months postoperatively, the wound was well-healed, and her catamenial thigh pain had greatly improved. As she had been treated with a GnRH agonist, a bone mineral density study was performed and demonstrated reduced bone density. Leuprolide acetate and norethindrone were discontinued, and she was referred to an endocrinologist for management and careful follow-up. Following counseling on the risks and benefits of various medical management options, dienogest 1 mg oral daily was prescribed for ongoing hormonal suppression. With this, her cyclic pain resolved and she remains pain-free 18 months postoperatively.

## Discussion

Extrapelvic musculoskeletal endometriosis has been rarely described (3–9). To our knowledge, this is the first case of musculoskeletal endometriosis in the lower limb to present following a history of pelvic fracture, prompting consideration of possible pathophysiologic mechanisms. Most commonly referred to theories to explain the development of endometriosis include Sampson's theory of retrograde menstruation (10) and the theory of coelomic metaplasia (11). While these theories can explain the development of intraperitoneal endometriosis, it is difficult to apply these theories when considering the occurrence of extrapelvic endometriosis at sites outside of the abdominal cavity. A less definitively proven theory is the idea that endometrial cells are disseminated *via* lymphatics or blood vessels (12) to distant locations.

The more recently proposed theory of endometriosis etiology is the stem or progenitor cell theory (13). Stem cells are undifferentiated cells that can differentiate into various cell lineages and characteristically are described to exist within the hematopoietic system. Evidence suggests that stem cells exist within the cycling endometrium, residing within the basalis layer (14). The data suggest that both uterine and bone marrow stem cells contribute to the development of endometriosis, and this may occur through mechanisms described in the previously discussed theories. For example, bone-marrow-derived stem cells may travel to ectopic locations *via* systemic hematogenous or lymphatic spread and differentiate into endometriosis (13). This theory is supported by the finding that musculoskeletal endometriosis can exist in the absence of pelvic disease (4–6, 8).

A review by Canis et al. (15) discusses the association between tissue trauma and the development of endometriosis. Specifically, it reviews *in vitro* studies that have demonstrated that endometrial fragments are more likely to adhere to locations where mesothelial cells are damaged or absent. Growth factors released in traumatized tissue may facilitate the implantation and growth of endometriosis (15). Uniquely, the patient has a history of left-sided pelvic fracture that preceded symptom onset. Given the location of the lesion of the patient, we consider whether retrograde menstruation tracked along the round ligament, through the inguinal canal, or toward the left labia. At the time of tissue trauma, these cells may have tracked along tissue planes toward damaged tissue in the medial thigh and developed into an endometriotic lesion. Alternatively, at the time of the left-sided pelvic fracture stem/progenitor cells within the bone marrow may have been released and tracked along tissue planes to adhere to traumatized tissue in the medial thigh. One other reported case of musculoskeletal endometriosis in the vastus lateralis documents a history of trauma that preceded symptom onset. In this case, the patient noticed the mass soon after sustaining a straining injury to her left thigh while lifting weights. The timeframe that would be expected to exist between endometriosis seeding and symptom onset is unclear. The patient developed symptoms approximately 11 years following a pelvic fracture. We suspect the rate of proliferation of the endometriotic lesion of the patient and her degree of symptom severity may have been influenced by the OCP which she had been taking in the previous years. This medication was likely insufficient to control her pain symptoms over the past 4 years.

This case presents similarly to other documented cases as a new painful mass or bulge in a muscular compartment with menstrual exacerbation (3–6, 8, 9, 16). While one previously reported case documents the presence of pelvic endometriosis following laparoscopic excision of ovarian endometriomas (9), most patients with the musculoskeletal disease do not have a documented pathologic history nor do they present with symptoms or radiographic evidence of pelvic endometriosis (4–6, 8). We did not definitively determine whether the patient had pelvic endometriosis, as pelvic symptoms were not a primary concern and laparoscopy was not indicated. In a case of knee endometriosis reported by Patel et al. (8), a diagnostic laparoscopy was done to look for pelvic endometriosis following a positive biopsy of the lateral knee mass and was found to be negative. Thus, pelvic endometriosis is not a prerequisite for ectopic musculoskeletal disease, and this diagnosis should be considered in all reproductive-age women presenting with catamenial muscular pain regardless of the known history of pelvic endometriosis. Although the patient originally presented with a recurrent Bartholin's abscess, we believe this to be a coincidental occurrence and a separate disease process from the musculoskeletal endometriosis, given that the catamenial musculoskeletal pain predated the Bartholin's abscess by 4 years and the abscess presented acutely and eventually resolved with drainage.

The reported management of extrapelvic musculoskeletal endometriosis is largely surgical excision (4, 6, 8, 9, 16). Before surgery, the patient trialed a GnRH agonist and experienced no symptom relief with regards to her catamenial thigh pain. Not all patients with traditional pelvic endometriosis respond to GnRH agonists (17), and these medications are not known to change the size of endometriotic implants. In this case, the severity of the disease of the patient as measured by size, fibrosis, and impingement on surrounding structures may have accounted for her symptoms and lack of response to treatment. One case of sciatic nerve endometriosis was treated exclusively with a GnRH agonist with complete resolution of symptoms (18), and the second case of gluteal endometriosis noted some improvement in symptoms with GnRH agonist treatment but ultimately surgical excision was required (19). These data suggest that a trial of medical treatment is reasonable with appropriate counseling and monitoring of response. In this case, the described surgery was performed by gynecology in consultation with a surgeon with musculoskeletal expertise. Similarly, Fambrini et al. (9) describe a case of leg adductor endometriosis in which orthopedic-oncology performed a surgical resection in conjunction with gynecology. A multidisciplinary approach may be considered in cases of musculoskeletal endometriosis to ensure optimal margins and to minimize morbidity secondary to surgical resection and defect closure.

Postoperative hormonal therapy was used in this case to achieve full symptom control, and this has been employed in several previously reported cases (2). It is unclear whether the residual pain of the patient 3 months after surgery was secondary to a prolonged healing course, given her postoperative wound complication, or secondary to residual microscopic endometriosis. The resolution of her pain following the initiation of dienogest supports the latter scenario. Guida et al. (19) describe a case of gluteal endometriosis wherein a patient continued to have some symptoms following surgical excision. One year postoperatively, an MRI revealed several small endometriotic foci likely representing residual disease. Unfortunately, the patient did develop reduced bone mineral density postoperatively which is a known side effect of leuprolide acetate and dienogest. We recognize that ideally no adjuvant hormonal suppression is required in these cases, although the experience indicates that patients should be counseled regarding the possible need for ongoing medical management postoperatively (20) and implications of this treatment.

In conclusion, extrapelvic musculoskeletal endometriosis is a rare entity with likely multifactorial pathogenesis. We are unable to assume causality of previous trauma on the development of extrapelvic musculoskeletal endometriosis in the lower limb; however, the association in this and another reported case prompts us to consider whether previous musculoskeletal trauma leads to the spread of stem/progenitor cells to ectopic locations. In addition, we discuss the possibility of retrograde menstruation along the round ligament and endometrial cell migration into the thigh following tissue trauma. Clinicians should inquire about catamenial exacerbation in any reproductive-age women presenting with painful musculoskeletal lesions irrespective of a known history of pelvic endometriosis. Diagnosis is achieved *via* a combination of clinical history, physical examination, imaging, and possibly a biopsy. Management of this condition is largely surgical excision, although postoperative hormonal suppression may be required.

## Patient Perspective

“I was active in a very physical sport. I started to have pain, and it was thought I had pulled my groin.” The injury never seemed to heal after many months. I followed up with my family doctor, who sent me for an ultrasound, which discovered a crack in my pelvis. No treatment was available for this, as it had already begun to heal.

My menstrual cycles became extremely heavy and painful while the mass grew extremely painful and debilitating at times. It became a nightmare for me to have a menstrual cycle and I was told by my family doctor this was normal. Some days I could not walk properly, I would have to stand at my office job, sit sideways, or on a pillow that caused back pain. I could not drive some days, as sitting was far too painful. I was on a steady diet of Advil and Tylenol. I used every muscle cream you could buy just to try to get some relief from the pain. I was no longer able to play any sports, exercise, or even just sit normally. It got to the point where I could no longer have intercourse with my spouse or insert a tampon. My family doctor sent me to the emergency room, and my first Bartholin's abscess was discovered. A drain was inserted. A month later, I had a second Bartholin and again another drain was placed. At this point, my OBGYN sent me for the first MRI, where the tumor was discovered.

There were points that I thought I was just going to have to live my life with the pain that I was experiencing. I was at the point where I was tired of being sick and in pain constantly. I am so thankful for the team of doctors that I had in my corner as I am now fully healed now and no longer have any lingering pain from the tumor. “The area where the tumor was removed is completely numb now, and they are not sure the feeling will ever come back, which I knew was a possibility; however, the relief of not being in constant pain is worth it.”

## Data Availability Statement

The original contributions presented in the study are included in the article/supplementary material, further inquiries can be directed to the corresponding author/s.

## Ethics Statement

Ethical review and approval was not required for the study on human participants in accordance with the local legislation and institutional requirements. The patients/participants provided their written informed consent to participate in this study. Written informed consent was obtained from the individual(s) for the publication of any potentially identifiable images or data included in this article.

## Author Contributions

All authors were involved in the care of the patient described in this case and contributed to manuscript development and revision.

## Conflict of Interest

The authors declare that the research was conducted in the absence of any commercial or financial relationships that could be construed as a potential conflict of interest.
